# The Social Health Intervention Project (SHIP): Protocol for a randomized controlled clinical trial assessing the effectiveness of a brief motivational intervention for problem drinking and intimate partner violence in an urban emergency department

**DOI:** 10.1186/1471-227X-14-10

**Published:** 2014-04-18

**Authors:** Karin V Rhodes, Melissa Rodgers, Marilyn Sommers, Alexandra Hanlon, Paul Crits-Christoph

**Affiliations:** 1 Perelman School of Medicine, University of Pennsylvania, Philadelphia, USA; 2 School of Nursing, University of Pennsylvania, Philadelphia, USA; 3 Center for Emergency Care Policy Research, Department of Emergency Medicine 1st Floor Ravdin, HUP, University of Pennsylvania, Philadelphia, PA 19104, USA

**Keywords:** Problem drinking, Motivational interviewing, Intimate partner violence, Emergency department, Screening brief, Intervention and, Referral to treatment

## Abstract

**Background:**

There is a strong reciprocal association between two highly prevalent public health problems: intimate partner violence and heavy drinking, both of which remain major sources of morbidity and mortality. Brief interventions in the Emergency Department setting have been found to be effective in reducing alcohol-related injury but neither classic intimate partner violence nor substance abuse interventions have adequately integrated assessment and treatment for these co-occurring conditions. The overall goal of this study is to determine whether a motivational intervention delivered at the time of an Emergency Department visit will reduce heavy drinking and improve the safety of women experiencing intimate partner violence.

**Methods and design:**

We are completing data collection for a randomized controlled trial enrolling 600 female patients, age 18–64, presenting to one of two urban Emergency Departments, who self-disclose both problem drinking and intimate partner violence. Eligible patients are randomized to a brief manual-guided motivational intervention, and a phone booster at 10 days. The intervention, which is delivered by masters-level therapists during the Emergency Department visit, is recorded and monitored for fidelity. Primary outcomes are episodes of heavy drinking and incidents of intimate partner violence, assessed weekly by Interactive Voice Response System for 12 weeks and at 3, 6 and 12 months by interviewers blinded to group assignment. To identify the impact of assessment alone, we included a no-contact control group assessed only once at 3 months. Secondary outcomes include violence severity, changes in the Composite Abuse Scale and alcohol quantity/frequency, along with other health-related behaviors. The analysis will also explore the impact of likely mediators and moderators of the intervention.

**Discussion:**

While screening and intervention for intimate partner violence is now recommended for women of child bearing age in health care settings, there is a need for rigorous evaluations of what works for whom. Upon completion, we will have high-quality evidence regarding the effectiveness of a low-intensity, brief motivational intervention, delivered by social workers in the Emergency Department setting, for decreasing episodes of heavy drinking and intimate partner violence. Ultimately, this is a model could be generalizable to other acute health care settings.

**Trial Registration:**

ClinicalTrials.gov Registration Number: NCT01207258

## Background

The purpose of this paper is to present the details of the protocol being used in a large randomized clinical trial to test the effectiveness of a brief motivational intervention, delivered by trained and supervised masters-level therapists to women experiencing intimate Partner Violence (IPV) and problem drinking at the time of their visit to an urban Emergency Department (ED).

IPV is defined as physical, sexual or psychological harm perpetrated by a current or former intimate partner. Problem drinking includes the full spectrum of harmful, hazardous, or heavy drinking as well as alcohol dependence. These two public health concerns are highly correlated and present major costs to individuals and societies [1-5]. IPV is a major source of morbidity and mortality, with one in four women and one in seven men in the U.S. experiencing physical, sexual, and/or psychological abuse by an intimate partner in their lifetime [6]. The health consequences of IPV include high rates of injury, chronic pain, anxiety, depression, somatic concerns, and substance abuse [7-10]. Likewise, approximately three in ten U.S. adults drink enough to increase their risk of physical, mental health, and social problems, and about 25% of these at-risk individuals currently experience alcohol abuse or dependence [11]. Problem drinking is strongly correlated with a variety of injuries [12], and an estimated 7% of all ED admissions are alcohol related [13]. A recent analysis of the 2003 National Hospital Ambulatory Medical Care Survey (NHAMCS) found that patients with alcohol-related injuries require excess of procedures, tests and monitoring during their ED visit, and are two times more likely to be admitted to the hospital [14]. The Centers for Disease Control estimate the cost of excessive alcohol consumption in the US to be $224 billion, with three-quarters of the cost related to binge drinking [15].

There is some evidence that counseling interventions for IPV that also target alcohol use can result in a decrease in both risks [16]. To date, these studies have primarily focused on couples and the male drinker, and the findings have not been successfully translated into individual interventions for the female partner. Likewise, interventions for IPV-involved women have yet to incorporate evidence-based interventions for alcohol abuse [17]. IPV and heavy drinking have both been identified as significant risk factors for each other [18]. The complexity of the relationship between heavy drinking and IPV may make it difficult for interventions to adequately address one risk without considering the other. The current study addresses the need for evaluating interventions that target comorbid risks of heavy drinking and IPV in real world settings.

Ideally suited to the fast-paced ED setting, brief motivational interventions for alcohol have been found to be effective at reducing problem drinking among male patients but results have been inconsistent among women [19]. The Gentilello et al. study [20], with patients hospitalized after a serious alcohol-related injury, found that, compared to controls, a brief motivational intervention was effective in men but not women. They attributed the gender difference to women drinkers’ higher rates of psychosocial and relationship problems, specifically recent abuse. The authors recommended that brief alcohol intervention programs need to have the capacity to address these co-occurring issues in women. In contrast, Blow et al. [21] found that a sub-group of young college-age women (ages 19–22) who received brief advice were the group most likely to reduce their frequency of binge drinking.

A Cochrane review [19] of brief motivational interventions in primary care – a meta-analysis of 22 RCTs with 7,619 participants – found that participants receiving a brief motivational intervention had lower alcohol consumption than control groups on follow up of one year or longer. Meta-regression did not find evidence of greater reduction in alcohol consumption with longer treatment exposure or among less clinically representative samples. Important to the current proposal, the Kaner Cochrane [19] review specifically states that the benefit of brief motivational interventions for problem drinking in women is not clear, and recommends "future trials should focus on women and on delineating the most effective components of interventions" (p.2). The question of whether or not the mixed results by gender found with motivational interventions are due to unmeasured IPV among women drinkers remains unanswered. Neither IPV nor substance abuse interventions have integrated assessment and treatment for these co-morbid risks. There are no studies evaluating the long-term impact of IPV interventions for IPV-involved women drinkers in health care settings. Based on the available research, we conclude that the current study will fill an important gap in the literature by evaluating the potential of a brief motivational intervention in an acute care setting.

### Study aim

**Figure 1:**
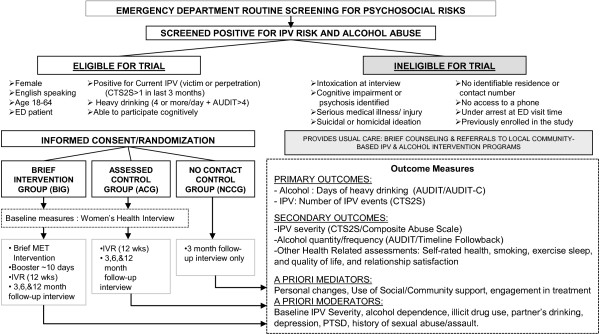
Study flow chart.

This study intends to evaluate the effectiveness of a manualized, low-intensity, gender-sensitive, brief motivational intervention to alleviate two strongly associated risks, relationship violence and heavy drinking. Specifically, the study team aims to decrease instances of IPV (both victimization and perpetration) and episodes of heavy drinking (4 or more drink on any one occasion) among IPV-involved women who are heavy drinkers (See Figure 1).

### Objectives

● To determine if a brief ( 20-25 minute) manual-guided motivational enhancement intervention, which could be generalizable to other acute health care settings, is effective at decreasing instances of IPV and days of heavy episodic drinking among female patients in urban ED settings who report IPV in the past 3 months and heavy drinking.

● To assess the impact of the brief motivational enhancement intervention on IPV severity, alcohol consumption, self-rated health, health behaviors, quality of life and relationship satisfaction.

● To determine the impact of likely moderators of the intervention’s effect, such as the baseline level of IPV severity, alcohol dependence, co-occurring illicit drug use, depression, PTSD, child history of sexual abuse, and partner drinking.

● To explore potential mediators of the effectiveness of the intervention, such as changes in self-efficacy, readiness to change, social support, and follow up with alcohol or IPV services.

## Methods and design

### Setting, timeline, and general study design

This study is a longitudinal, randomized controlled trial, being conducted in two urban academic EDs staffed by emergency medicine faculty, residents, and nurse practitioners. These are the primary EDs and trauma units serving a 14-square mile area with mixed socioeconomic status and a population of over 200,000. Using a conservative prevalence range of 5-10% for risk of both IPV and problem drinking, we anticipated that recruitment from this medical venue would be adequate to achieve our target sample size based on the high patient volume (approximately 1,535 women subjects/year in the target age range). and the tendency for IPV victims and problem drinkers to receive their medical care from EDs The inclusion of two locations broadens our population and allows for greater generalizability.

Participants are randomized into one of three study groups using a 2:2:1 distribution: the intervention group (n = 240), the assessed control group (n = 240) and the no contact control group (n = 120). The assessed control group is interviewed at enrollment and at similar time points to the intervention group. The no contact control group is only assessed at 3-months, which enables us to determine the extent to which improvements over time in the intervention and assessed control groups maybe due to assessment reactivity.

This RCT began enrollment on January 18, 2011 and the target enrollment of 600 women was completed earlier than planned on November 6, 2013. Currently, we are in the middle of follow up data collection. Data collection will continue until approximately December 2014, the point at which all women in the intervention and assessed control group will have complete 12 month follow up measures.

### Conceptual model

**Figure 2:**
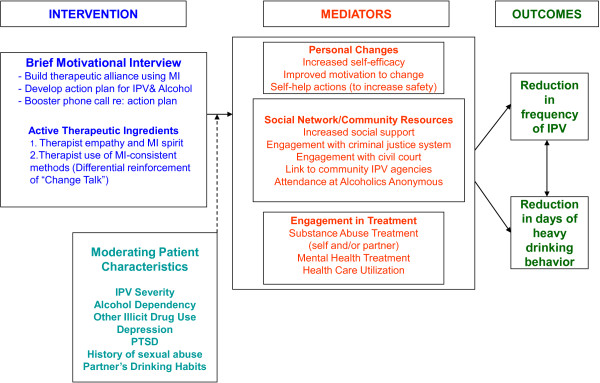
Conceptual model.

As depicted in Figure 2, we expect that the magnitude of the intervention’s impact will be influenced by several moderating patient characteristics and mediating factors. Our identified mediators are potential post-intervention changes in personal, social/community, and treatment utilization factors that we hypothesize will contribute to and support reduced IPV frequency and reduced days of heavy drinking. In addition, Figure 2 also reflects our recognition that the level at which a patient is able to initiate positive change related to the personal, social, and treatment-based aspects of her life may depend on several moderating patient characteristics. It is possible, for example, that a patient’s depression (moderator) may impede her ability to take steps to increase safety (mediator) and therefore will reduce the effectiveness of the intervention on IPV frequency (outcome) when compared with a patient who is not depressed.

### Study hypotheses

● At-risk women who receive a brief motivational intervention – compared to an identically-assessed usual care group will have a reduction in days of heavy drinking (4 or more drinks/day) and incidents of IPV-assessed weekly for 3 months and by follow up interviews at 3, 6, and 12 months.

● Women’s self-efficacy, readiness to change, and rates of engagement with informal and formal social support systems will mediate the effect of the intervention on our primary outcomes.

● PTSD, a history of childhood sexual abuse, higher baseline severity of IPV and dependent drinking will moderate or reduce the effectiveness of the brief intervention.

### Inclusion criteria

Patients must meet the following criteria to qualify for study participation at the time of assessment:

● Female patients ages 18–64

● Able to participate verbally and cognitively in an English language interview

● Heavy drinking (4 or more drinks on at least one day; 7 or more drinks in one week)

● A positive screen for IPV by a current or former partner in the past 3 months

### Exclusion criteria

● Intoxication at the time of screening

● Cognitive impairment or psychosis identified on physical examination or chart review

● Serious current medical illness or injury, defined as respiratory distress, hemodynamic instability, active vomiting, bleeding, labor, severe pain, or acute need for hospital admission

● Suicidal or homicidal ideation by chart review

● No identifiable residence or contact phone number

● Under arrest at the time of ED visit

● Non-English speaking

● Previously enrolled in the study

### Screening and subject recruitment

Data collection occurs approximately 10 hours per day, 6 days each week and ED providers are encouraged to make referrals of potentially-eligible patients during off hours. During data collection periods, Research Assistants (RAs) approach and attempt to screen all female patients in the ED using a psychosocial risk assessment (referred to as the Social Health Survey) that asks about IPV and risky drinking. Patients who indicated a risk for either IPV or risky drinking are asked to complete a formal assessment to determine study eligibility. The assessment includes the AUDIT [22] and CTS2S [23], is verbally administered by the RA to determine study eligibility of IPV and excessive drinking in the last 3 months – a score of 1 or more on the CTS2S and 4 or more on the AUDIT. For referrals outside of data collection hours and for women who are found to be potentially eligible but unable to stay in the ED, the Clinical Research Coordinator collects safe phone contact information to determine study eligibility and arranges an appointment for enrollment.

### Consent & randomization

Prior to consent, patients are informed that the enrollment process could take up to an hour to complete, whether or not they are discharged from the ED. Using the SPSS pseudorandom number generator [24], the study statistician created a block randomization scheme, in groups of 20, to determine study assignment. The project manager used the scheme to prepare two sets of opaque envelopes – one for each ED – that are indistinguishable from each other and thick enough so that their contents are not legible from the outside. All patients who give consent for participation are randomized by pulling the next sequential envelope. This process ensures that those who enroll participants are unaware of group assignment until after they are consented to be in the study; only the project manager and statistician are aware of the randomization scheme. For patients randomized to the intervention group, the RA presents a secondary consent for audio-recording of the motivational interview. Participants do not have to consent to audio-taping to participate in the study.

### Data collection instruments

#### Medical record data and the social health bin

**Figure 3:**
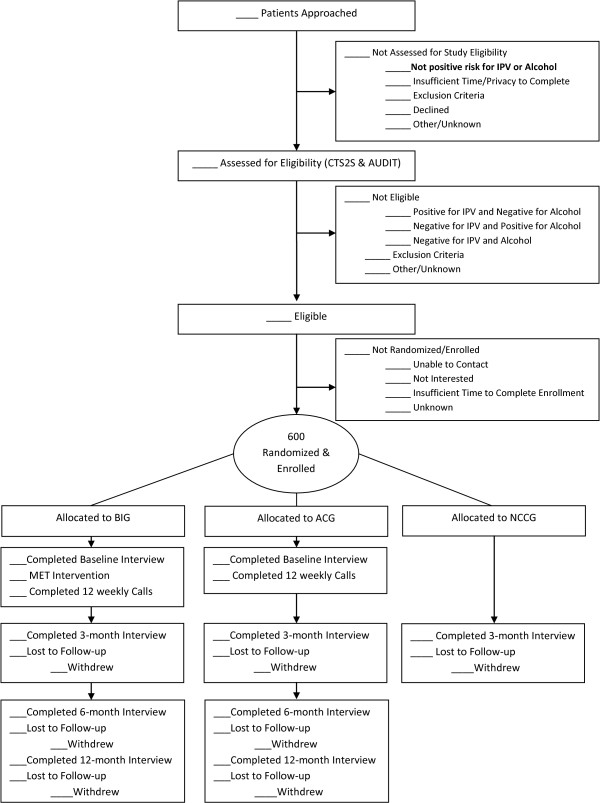
CONSORT flow.

Using the ED electronic medical record system EDs that captures patient demographics, medical history, and visit information, the research team developed a Social Health Bin, an electronic view of current patients filtered by demographic inclusion criteria (female, ages 18–64). Only study staff have access to the bin, which RAs use to (1) identify patients that meet study demographic criteria and (2) electronically document all engagement information for all tracked patients, e.g. whether patients are screened, assessed, offered and accept or decline enrollment into the study. This information can be exported from the system along with key visit information (i.e. chief complaint, triage level, insurance). These data will be used to construct our CONSORT flow, as it includes reasons why patient were not screened, assessed, or willing to enroll (See Figure 3). It will also allow us to compare demographic information between the populations of ED women who are enrolled vs. not enrolled in the study.

#### Participant demographic form

This data collection form is collected from all study participants at enrollment and includes detailed personal information, such as relationship status, household income, and education level.

### Structured women’s health interview

The women’s health interview is an extensive assessment, which includes a variety of validated measures for secondary outcomes, mediators and moderators (See Additional file 1: Table S1). Participants in the intervention and assessed control group are administered the interview at baseline, 3 months, 6 months, and 12 months. The no contact control participants only complete the interview at 3 months. All interviews are structured with close coded responses to validated questionnaires and take approximately 30 minutes to complete. Follow up interviews can be conducted over the phone or in person. RAs conducting follow up interviews are blinded to the participant’s group assignment during data collection. At the completion of all data collection, control group participants are offered an opportunity to receive the intervention, which is usually scheduled as a phone session by one of the motivational therapists.

### Interactive voice response system

Participants in the assessed control group and intervention group complete a weekly phone survey measuring frequency of abuse or violence in their relationship, and consumption of alcoholic beverages. The first call is conducted in the ED at the time of enrollment. Participants call the toll-free number, which is open twenty-four hours a day, seven days a week, using a patient number and unique password to protect confidentiality. Prior to entering the password, anyone who calls the telephone number for the survey will hear only a generic message that does not refer to a study of IPV or alcohol.

### Retention

The clinical coordinator contacts participants by phone for follow up interviews, following an Individualized Safe Contact Plan that the participant helps to develop at baseline and is updated at every contact. This form includes participant preferences for follow up calls and secondary contact information in the event the participant cannot be reached. All attempted contacts and interactions are documented on the paper Study Participation Log and in the electronic database. Participants are contacted two weeks prior to their 3, 6, 12 month follow up date, and asked to schedule a time to complete their interviews. This lead time is important as it allows for multiple contact attempts prior to reaching the data collection time point. Once an appointment is made, the coordinator sets a reminder to make a confirmation and reminder phone call 1–2 days before the scheduled date.

The project manager tracks completed Interactive Voice Response System surveys. If a participant misses their first call outside the ED, or any two consecutive calls, the clinical coordinators contact the participant to remind them to complete her calls and determine if she still has all the information to complete the call.

Secondary contact persons (friends and/or family) who are identified by the participant during the creation of the Safe Contact Plan may be contacted if (1) the participant’s phone is no longer in service for over 2 weeks and/or (2) the clinical coordinator, project manager, or the motivational therapist are concerned about the participant’s safety. Research personnel never share information regarding the nature of the study with these contact persons; rather the goal is to inquire about alternative contact information for the participant.

Due to high ED recidivism rates in the study population, an electronic flagging system was developed to quickly identify returning study participants and prevent double enrollments. A green check appears next to a patients name in the Social Health Bin when an enrolled participant returns to the ED. If a green check shows up in the bin, the RA immediately notifies the coordinator to determine whether the participant is due for any follow up activities. In addition, the RAs collect new safe contact plans from all actively enrolled patients, regardless of whether she is due to complete any study activities. This method of contact is particularly important for our transient and difficult to reach study population. Safety concerns for study participants who are in abusive relationships limit our methods of contact to those approved by the patient on their safe contact form; meaning we cannot physically show up at their home or work or other methods used in less sensitive studies to increase retention.

### Intervention

#### Motivational enhancement therapy

The Motivational Enhancement Therapy (MET) intervention is a short, 20–25 minute counseling session that incorporates brief feedback and guidance with motivational enhancement techniques to assist patients in increasing their safety. The MET intervention primarily employs Motivational Interviewing techniques; however, it deviates from pure motivational interviewing by incorporating a feedback component. Previous studies have documented the importance of this component [25]. As with Motivational Interviewing, the goal of an MET intervention is to elicit the client’s self-identified reasons for change (not the practitioner’s) and help the client identify their own goals and resolve ambivalence.

Prior to project funding, the research team developed, piloted, and revised a brief MET manual. The manual derives from previous motivational intervention manuals targeting drinking and risky driving, adapted for ED settings [26-29]. Our manual was developed with input from Motivational Interviewing expert, Theresa Moyers, developer of the Motivational Interviewing Treatment Integrity (MITI-3.1.1 rating scale) [30], which is the adherence measure used in our study. The manual is available on request from the principal investigator, Karin Rhodes, MD MS (Contact email: Karin.Rhodes@uphs.upenn.edu).

During the MET interventions, which are recorded and analyzed for fidelity, participants are encouraged to identify any linkages between their drinking habits and their involvement in partner violence. For many, the outcomes of the MET intervention will be an agreement to reduce either alcohol use or its ability to cause harm (medical problems or trauma), to identify and agree to implement effective coping strategies for situations that are high-risk for IPV, and/or a connection with informal or community-based supports via therapist referral. The practitioner and patient come to this agreement through a process of negotiation. If the patient expresses an unwillingness or inability to consider change, the MET therapist’s primary role is to encourage the patient to explore any existing ambivalence and to support the patient’s autonomy and personal agency, even if she makes a decision not to change. In MET, the spirit of preserving and supporting the client autonomy and personal choice is paramount [31,32].

This intervention includes a (non-recorded) phone follow up booster component, which is intended to consolidate and reinforce the MET session. Longabaugh [32] found that a booster session 7 to 10 days after the ED visit resulted in significant reduction in alcohol-related negative consequences and alcohol-related injuries as compared to standard care and a brief intervention group without booster.

### MET therapists

The MET therapists, who work in an “on-call” capacity, are Masters-level counselors, with degrees in psychology, social work, or a closely related field and prior clinical experience. All MET therapists have experience working with abused women and/or receive formal domestic violence training. METs therapists are trained for two days on using the treatment manual by a Motivational Interviewing specialist who is a member of the Certified Network of Motivational Interviewing Trainers. Prior to conducting an MET session with a study participant, the MET therapist conducts 2–3 practice sessions with ED patients – focusing on IPV and/or alcohol consumption. Practice tapes are rated for adherence by the Motivational Interviewing clinical supervisor. MET therapists are only added to the on-call schedule once they are consistently adherent to the intervention. Additionally, MET therapists receive ongoing clinical supervision from a doctoral-level social worker trained in Motivational Interviewing. Supervision occurs weekly, in a group format. During supervision, recordings of MET sessions are reviewed for adherence to the MET protocol. Motivational Interviewing expert, Theresa Moyers, PhD, reviews a randomly selected 10% of all audiotapes, to provide on-going monitoring for adherence to the treatment manual. After study enrollment is complete, all recordings will be sent to Dr. Moyers’s team of PhD-level Motivational Interviewing experts for full adherence ratings, along with reliability and inter-rater reliability calculations.

### Research assistants: training & supervision

Research Assistants (RAs) are part-time research employees who work in the EDs during data collection periods. Their primary responsibilities include screening, assessing for eligibility, consenting and enrolling patients, administering the demographic form and the Women’s Health Interview, and enrolling participants in the Interactive Voice Response System. RA training involves human subjects certification, in-depth review of the study protocol, and role-playing all components of the study engagement and enrollment process. All staff must attend a 3-day intensive training that covers IPV safety issues and reviews project background and procedural information. When ready for the ED setting, they first observe/shadow a senior RA and then conduct study and enrollment procedures under the observation of the clinical coordinator prior to screening on their own. On-going periodic shadowing by the supervising on-site coordinator ensures rigorous fidelity to the study protocol, as well as regular performance feedback from the project manager. RAs attend bi-weekly meetings with the coordinators, project manager and PI, where they share their experiences, problems, and brainstorm solutions. Refreshers on specific study procedures also occur during these regular staff meetings.

### Data management and quality assurance

The data collection tools for this study are varied; however, all study outcome data is stored and entered electronically using REDCap. REDCap is a secure web-based application designed to support data capture for research studies, providing an intuitive interface for validated data entry and automated export procedures for seamless data downloads to common statistical packages [33]. In addition, all databases are password-protected, stored, and backed up daily on a protected server.

The Data Manager and statistician developed a duplicate data entry plan for all collected study data. Every six months, a comparison of the data entered in the Initial and Duplicate databases is conducted using SAS Proc Compare report [34] and then research staff manually review inconsistent observations using the paper case files. An error rate is calculated using all founded inaccuracies in the Initial database. All inaccuracies are corrected in the initial database. Since primary outcome data is located throughout the data collection tools, 100% of case files are entered into the Duplicate database and compared to determine if the error rate is above 5%, the threshold for additional double data entry. To date, the error rate is less than 1%.

### Statistical analysis

#### Analysis across all three groups

With a goal of identifying the impact of assessment alone, the outcomes below can be analyzed across all three groups: the Brief Intervention Group (BIG), the Assessed Control Group (ACG) and the no contact control group (NCCG) using a intent-to-treat analysis that includes all participants who complete randomization and enrollment procedures and provide baseline and/or at least one follow up data collection point. Analysis across all groups including the no contact control group (NCCG) will include eligibility assessment data (baseline) and 3-month interview data collected on CTS2S and the AUDIT assessments.

Alcohol outcome measures are the frequency of heavy drinking (4 or more drinks/day for women) collected at baseline in person and by phone interview at 3 months using one item on the AUDIT [22], to calculate the frequency of 4 or more drinks. The full AUDIT score (0–40) [22] and additional drinking data are collected using a calendar-based Timeline Followback [35] for the month (28 days) prior to the interview.

IPV outcome measures include the frequency of IPV incidents (emotional, physical, and sexual) collected at baseline and by phone interview at 3 months using the CTS2S [23], scored 0–96. These data are on a likert scale, capturing a range of frequencies of occurrence in the previous three months. IPV Severity is calculated as the number of IPV incidents classified as severe in the preceding 3 months measured using specific items on the CTS2S [23]. IPV type and severity is also captured through the Composite Abuse Scale [36], scored 0–150.

All of the above measures are collected at baseline and 3, 6, and 12 months for the Intervention and Assessed Control Group and at 3 months only for the Non-Assessed Control Group. So any comparison across all 3 groups is limited to 3 month outcomes.

Using the intent-to-treat analysis for all patients who complete enrollment as described above, we will rely on generalized linear modeling to examine baseline and 3-month responses, adjusting for any other covariates that are determined to be significant in the preliminary analyses. Through pair-wise comparisons, this analysis will also compare 3 month differences between the assessed control group and no contact control group to determine the impact of assessment on the outcomes. For the analyses of the impact of assessment bias, only participants with 3-month outcome data will be included analysis.

### Analysis of intervention and assessed control groups

We will compare the Intervention and Assessed Control Groups on primary outcomes of heavy drinking and incidents of IPV collected via the interactive voice response system for 12 weeks after enrollment and by interview at 3, 6 and 12 months.

### Primary outcomes collected by the interactive voice response system

1. **Days of Heavy Drinking:** For this comparison, the AUDIT-C [25] heavy drinking question is asked, but is captured in the Interactive Voice Response System as the number of days when 4 or more drinks were consumed in the last week.

2. **Number of IPV events:** The 8 CTS2S [23] victimization questions are used to measure the incidence of IPV, and counts are collected for these questions each week via the interactive voice response system.

Our primary analytic method for the two group longitudinal comparisons will include implementing Hierarchical Generalized Linear Models (HGLM) to accommodate the clustering of the weekly repeated measures, as well as the nature of the outcome, which counts the number of heavy drinking days and the incidences of IPV per week. If the outcome measure displays a large proportion of zeroes, we will implement zero-inflated Poisson or zero-inflated Negative Binomial models, as appropriate with random effects. These models accommodate the clustering as well as the stack of zeroes present across the repeated assessments. [38] To assess the sensitivity of treatment effect estimates to missing data, we propose to fit the hierarchical regression models without accounting for informative missing data, and then compare these results with those from analyses that adjust for informative missing data. We will employ the pattern-mixture approach as applied to hierarchical regression models by Hedeker et al (1997). [39] Their methods assess whether important terms depend on certain missing data patterns, and provide equations to obtain unbiased estimates by averaging over the various missing data patterns.

### Secondary outcomes

Secondary alcohol outcomes include changes in the full AUDIT [22] score and drinking data collected using a calendar based Timeline Followback [35] for the month (28 days) prior to the follow up interview. Secondary IPV outcomes include changes in the Composite Abuse Scale [36] and measures of the frequency and severity of IPV from the CTS2S.

Other health-related outcomes include: self-rated health, quality of life, sleep, and relationship satisfaction. For these secondary outcome measures collected at baseline, 3, 6, 9, and 12 months, Hierarchical Linear Models (HLM) will be implemented to compare the intervention and assessed control groups. Variables that show significant differences between the treatment groups at baseline will be included as covariates in the model.

### Main effects, moderator and mediator analyses

Predictor analysis will focus especially on baseline IPV severity collected using the Danger Assessment Scale [40], scored 0–39, the Women’s Experience with Battering [41] scored 10–40 and alcohol dependence as identified by an AUDIT [22] score greater than 13. We will also explore other plausible confounders and predictors identified in the literature including: the partner’s drinking; patient’s use of illicit drugs, depression, PTSD, and history of sexual abuse/assault as child and/or adult. These covariates will be analyzed in the same manner as the longitudinal analysis of the primary measure. For secondary efficacy measures collected at baseline, 3, 6, and 12 months, HLM models will be implemented to compare the intervention and assessed control groups. Potential predictors will analyzed both as main effects and potential interaction effects with group assignments to assess for moderation.

And finally, should the intervention group demonstrate significant differences on IPV and drinking outcomes, we will assess for changes in women’s self-efficacy, readiness to change, and rates of engagement with informal and formal social support systems or treatment as significant mediators of the effect of the intervention on our primary outcomes.

### Power for analyses

We conducted sample size estimation for analyses comparing among all three groups (intervention, assessed control, no contact control), with specific contrasts planned to compare (1) the intervention group to the no contact group, and (2) the assessed control group to the no contact group. Power analyses for these comparisons are based on mean differences at 3 months. With 120 randomized patients in the no contact control group, and 240 randomized to each comparison group, power (2-tailed; alpha = .05) is 80% to detect an effect size (Cohen’s d) of 0.33 with up to 30% attrition (84 vs. 168 patients for each comparison).

Additional power analyses were conducted for the comparisons of the Intervention and Assessed Control Group on the two primary outcome measures (reduction in heavy drinking; incidence of IPV). Based on previous studies [21,27], we considered a relative reduction of 2 or more (or approximately 35% reduction) heavy drinking episodes/month to be a clinically significant outcome. Blow [21] and D'Onofrio [27] suggest that rates of heavy drinking will be about 6.0 days/month (SD = 6.0) at baseline and end at about 3.5 days/month (SD = 5.0) at week 12 for interventions without a special motivational interviewing intervention that targets IPV and focuses on women only. For the power analysis given below we therefore specified a rate of change over weeks 4 to 12 in the brief intervention group (BIG) that will end with a 2 days/month improvement over the assessed control group (ie, 3.5 days per month for the assessed control group, 1.5 days/month for the brief intervention group).

In our calculations, we assume an autoregressive covariance structure with a within subject correlation of 0.6. The 0.6 assumption is comparable to what was seen with the Ball [42] study, where the average within subject correlation was 0.57. Converting the estimates above to weeks instead of months, assuming the effect seen in Ball [42] during the first month of treatment, we anticipate slope estimates per week during the second two months to be 0.109 and 0.046, respectively for the Assessed Control and BIG groups, with standard deviation over the second two months increasing proportionally from SD = 0 at week 4 to SD = 1.25 at week 12.

For the power analyses, we specified a Type-I error level (alpha-level) of 0.025 (splitting alpha between the two primary outcomes of heavy drinking days and IPV incidents). For heavy drinking outcomes, we derive a randomized sample size of 199 per group to achieve at least 80% power to detect a significant effect in the rate of change between groups with attrition/missing data of 30% during the first 12 weeks of treatment. For IPV primary outcomes, this size sample (199 per group) yields power of 93.7% for detecting a clinically significant result with 30% attrition/missing data across 12 weeks. Thus, a randomized sample of 240 subjects per group needed for the comparisons with the no contact control group is more than adequate for conducting the primary analyses comparing BIG to the Assessed Control groups.

For the analysis of intervention moderators, we assume that if the moderator variables are measured without error, we need a sample size of 55 to have 80% power to detect a medium effect with an alpha of 0.05. While demographic variables are measured without error, the baseline risk status variables have reliability indices closer to 0.90. Aiken and West [43] argue that the sample size required to reach a power of 80% with an alpha value of 0.05 is slightly more than doubled when reliabilities drop from 1.0 to 0.80. According to these standards, our sample size of 240 per group (accounting for 30% attrition) is more than sufficient to detect a medium effect size for a moderator.

### Safety and ethical considerations

The study is conducted in accordance with Good Clinical Practices and CONSORT guidelines, and has received ethical approval from the University of Pennsylvania Institutional Review Board (Protocol #809428). A NIH Certificate of Confidentiality was obtained prior to the launch of the project.

Due to the high-risk nature of the IPV-positive study population, several important safety considerations were incorporated into study procedures. During enrollment, a “safe contact plan” is created together by the RA and participant (See Additional file 2). This subject-specific plan collects in-depth information on how to safely reach the participant during study activities. Questions include best days and times to call, time periods when study staff should not call, and how research staff should identify themselves in voicemail messages and if another person answers the participants’ phone. Code words and next steps are also developed for scenarios under which the participant needs to hang up the phone, and if the participant needs study staff to contact the police. The safe contact plan is updated at every study activity and when participant contact information changes. A suicide ideation procedure and diagram were developed to guide study team members should a participant indicate potential current suicide ideation. Note, while suicidal or homicidal intent at baseline is an exclusion criterion prompting notification and assessment by the treating physician, if either risk is identified after a patient is already enrolled in the study, they will continue to be followed in the study. Our protocol includes additional assessments, which are tracked in the database, by the PI and/or a skilled mental health therapist who will help to facilitate mental health care.

### Data safety monitoring board (DSMB)

A DSMB was organized to monitor the progress of the study, and recommend modifying the trial or terminating the trial as appropriate. The committee is composed of three scientists who are independent of the study, and two of whom are independent of the investigators institution. The study statistician serves as a non-voting member of the DSMB, and one Co-Investigator serves as the chair of the DSMB. Concerns that might dictate modification or termination of the study by the DSMB include participant safety, outcome data (data quality, integrity, and intervention efficacy), recruitment, and performance. Well-defined stopping rules were established prior to the first meeting to guide expected causes of termination.

## Discussion

Although a relationship between heavy drinking and IPV is well documented, few interventions for the individual have targeted co-morbidity as a primary aim. Information on the efficacy of IPV interventions is lacking and there are no long-term evaluations of IPV interventions in health care settings. This study aims to fill these gaps by assessing whether a brief intervention that seeks to address both risks, in an acute care setting, can help reduce IPV and problem drinking among women. This dual aim augments previous explorations of brief motivational interventions by acknowledging the complex interaction of co-occurring conditions. These risks have major public health implications, both independently and in conjunction. Results from this study, regardless of outcome, will add to a growing body of literature on the feasibility and safety, appropriate outcome measures, and the effectiveness of IPV intervention studies in the acute care setting.

### Strengths & relevance

This study will also provide insight for future interventions seeking to implement rigorously monitored brief interventions in hectic urban EDs or other acute care settings. Throughout the design and implementation of this protocol, we attempted to address the various obstacles for conducting longitudinal research in an ED with this vulnerable population, such as: capacity building, stakeholder buy-in, and technical considerations. Learning from the successes and failures of previous research, we utilize a variety of safety measures, strategic retention procedures, and innovative technologies for incentive payments and remote data collection. The treatment manual and adherence measures and innovative methods of subject retention will be a public resource for researchers and advocates engaged with this vulnerable population.

## Abbreviations

AUDIT: Alcohol use disorder identification test; CTS2S: Conflict tactics scale short form; DSMB: Data safety monitoring board; ED: Emergency department; GENACIS: Gender alcohol and culture: an international study; HLGM: Hierarchical generalized linear models; HLM: Hierarchical linear models; IPV: Intimate partner violence; IVRS: Interactive voice response system; NIAAA: National institute on alcohol abuse and alcoholism; PTSD: Post traumatic stress disorder; RA: Research assistant.

## Competing interests

The authors declare that they have no competing interests.

## Authors’ contributions

KVR, PCC, and MS applied for the NIAAA grant, and provided the initial draft of the proposed protocol. AH and PCC wrote the Statistical Analysis Section. KVR and MR created the initial draft of the full study procedural manual and the initial draft of this manuscript. All authors contributed to the development of the study protocol and procedures, edited drafts of the manuscript, and read and approved the final manuscript.

## Pre-publication history

The pre-publication history for this paper can be accessed here:

http://www.biomedcentral.com/1471-227X/14/10/prepub

## Supplementary Material

Additional file 1: Table S1 Assessment Battery. [37], [44-53]. Click here for file

Additional file 2 Women’s health study individualized safe contact plan. Click here for file
