# Supplementary material for: The Social Health Intervention Project (SHIP): Protocol for a randomized controlled clinical trial assessing the effectiveness of a brief motivational intervention for problem drinking and intimate partner violence in an urban emergency department
Source: BMC Emerg Med. 2014 Apr 18;14:10. doi: 10.1186/1471-227X-14-10 (PMC4101846; doi:10.1186/1471-227X-14-10)
Supplement: Additional file 1: Table S1 — Assessment Battery. [37], [44-53]. [file 1471-227X-14-10-S1.docx]

**Table S1** Assessment Battery

| Variable | Construct | Measure | Measure Ref. | # items | Sample Item | Response Type |
| --- | --- | --- | --- | --- | --- | --- |
| Primary Outcomes | IPV Frequency | CTS2S | Straus & Douglas [23] | 16 | # times partner hit, punched, or beat up. | # incidents |
|  | Heavy Episodic Drinking | AUDIT/ | Reinert [22] | 3 | How often do you have 4+ drinks on one occasion? | # heavy drinking days |
|  |  | AUDIT-C | Bush [25] |  |  |  |
| Secondary Outcomes | IPV Severity (past wk.)  ·Victim ·Perpetrator | CTS2S | Straus & Douglas [23] | 6 | #times partner used force to have sex. | #categorized as "severe" |
|  |  | Compositive Abuse Scale (CAS) | Hegarty et al. [26] | 30 | How many times in the past 3 months have the following things happened to you: | Likert (0–5) |
|  |  |  |  |  | - Told me that I was ugly. |  |
|  |  |  |  |  | -Tried to keep me from seeing or talking to my family. |  |
|  | Alcohol quantity/ frequency (past wk.) | AUDIT-C | Bush [25] | 3 | In the past week, how many days have you had a drink containing alcohol? | #days/week X #drinks/day |
|  |  | Timeline Followback | Sobell & Sobell [27] | 28 days | Please indicate the number of drinks you had on each day for the past month (28days) | Calendar documentation of number of drinks |
|  | Self-Rated Health |  | Andresen et al. [28] | 1 | How would you say your health has been? | Likert (1–5) |
|  | Health Behaviors ·Smoking ·Exercise | GENACIS (5 selected items) | Wilsnack & Wilsnack [29] | 2 | How many days/week do you engage in vigorous physical exercise? | #cigarettes #days exercise |
|  | Quality of Life | WHOQOL Qs | WHO [30] | 3 | How much do you enjoy life? | Likert (1–5) |
|  |  | (3 selected items) |  |  |  |  |
|  | Relationship Satisfaction | Dyadic Adjust. Scale | Hunsley et al. [31] | 1 | Which response best describes the happiness in your relationship? | Likert (0–6) |
| Mediators |  |  |  |  |  |  |
| Personal Changes | Self-Efficacy | Generalized Self Efficacy Scale | Schwarzer [32] | 10 | I can always manage to solve difficult problems if I try hard enough. | Likert (1–4) |
|  | Motivation to Change importance/confidence for alcohol/IPV | Importance/ Confidence Rulers | D’Onofrio et al. [33] | 4 | On a scale of 1–10, how important is it to you to take steps to change the conflict in your relationship? | Likert (1–10) |
|  | Self-help actions | Steps to Safety |  | 1 | Have you taken any steps in the past 3 months to increase your safety? | Yes/No If Yes, Describe (qual.) |
| Social/Community | Social Support | Social Support Network Scale | Block [34] | 3 | Do you have someone you could stay with if needed? | Yes/No Score 0-3 |
|  | Engagement with: |  |  | 3 | Have you used any police or court services because of family violence? | Yes/No |
|  | ·Criminal/Civil Court |  |  |  |  |  |
|  | ·IPV agencies |  |  |  |  |  |
|  | ·Alcohol peer support |  |  |  |  |  |
| Engagement in Treatment | Outpatient/Inpatient: |  |  | 9 | How many times have you seen a health professional on an outpatient basis for problems with alcohol? | # treatment visits |
|  | ·Alcohol ·Drug |  |  |  |  |  |
|  | ·Mental Health |  |  |  |  |  |
|  | ·Healthcare |  |  |  |  |  |
|  | ·Significant Family Problems |  |  |  |  |  |
| Moderators | IPV Severity at Baseline (low/high) | Women's Experience with Battering Scale | Smith et al. [35] | 10 | I feel like he keeps me prisoner. | Likert (1–6)Score 10-60 |
|  | IPV Severity at Baseline (low/high) | Danger Assessment | Campbell [36] | 20 | Does he own a gun? | Yes/No Weighted score |
|  | Likely Alcohol Dependence | AUDIT Qs 4,5, 6 | Reinert [22] | 3 | How often have you needed a first drink in the morning to get going? | Likert (0–4) + or - for dependence |
|  | Illicit Drug Use | GENACIS (4 selected items) | Wilsnack & Wilsnack [29] | 3 | In the past 3 months have you used marijuana? street drugs? Misuse Rx drugs? | Yes/No & Frequency |
|  | Depression | CES-D10 | Radloff [37] Andersen et al. [38] | 10 | I was bothered by things that don’t usually bother me. | Likert (0–3) Score 0–30 (11+ = depression) |
|  | PTSD | Primary Care PTSD Screen | Prins et al. [39] | 4 | Have you had an experience that was so upsetting that you have had nightmares about it? | Yes/No Score 1–4 (Score 3+=PTSD) |
|  | Sexual Abuse History | GENACIS (2 edited items) | Wilsnack & Wilsnack [29] | 2 | Before you were 16 years old did someone try to make you do/watch sexual things? | Yes/No |
|  | Partner Drinking | GENACIS | Wilsnack & Wilsnack [29] | 6 | When you and your partner quarrel, how often has your partner been drinking? | Likert (1–5) All the time/Most times… |
