# Supplementary material for: The Social Health Intervention Project (SHIP): Protocol for a randomized controlled clinical trial assessing the effectiveness of a brief motivational intervention for problem drinking and intimate partner violence in an urban emergency department
Source: BMC Emerg Med. 2014 Apr 18;14:10. doi: 10.1186/1471-227X-14-10 (PMC4101846; doi:10.1186/1471-227X-14-10)
Supplement: Additional file 2 — Women’s health study individualized safe contact plan. [file 1471-227X-14-10-S2.pdf]

Appendix A

Women's Health Study

INDIVIDUALIZED SAFE CONTACT PLAN

Participant Name: ____

Last First Middle I.

Group Assignment: NCCG ACG BIG

Date of Contact Plan____/____/ _______ **If BIG, Name of MET:** _________________

□ Check Here if Participant does NOT want to be contacted during follow-up.

"Your safety is our primary concern. We need to make a safe plan for contact. We do not want to call when your partner is around or can listen in on the conversation."

Please provide information about the best ways and times to reach you."

1. What time do you prefer us to call you?

   Day (please circle): M T W Th F Sa Su Time (please circle): Mornings Afternoon Evening

2. Are there any times when we should **NOT** call you? Day (please circle): M T W Th F Sa Su

   Time (please circle): Mornings Afternoon Evening

3. Is it ok for us to send you generic health tip reminders to your home address? □ Yes □ No
4. Is it ok for us to send you text messages with study reminders using generic messages? □ Yes □ No

   Sample: "Penn Medicine cares about your health. This is a friendly reminder. Call to schedule your next visit: 215-573-3055. Do not reply"

Is it safe to leave a message from 'Women's Health Project'?

____

____

How should we introduce ourselves if someone else answers the phone (e.g. Women's Health Stury; SHIP project)?

____

____

"If you are speaking with us and it suddenly becomes unsafe, you can just hang up, or say anything you want. We will understand that you cannot talk anymore at that time. You do no have to explain. If this happens, would it be okay for us to call you again later? How should we reach you? Do you want to use particular code words to indicate that it is not safe for you to continue the call at that time? Please provide any information that might be helpful."

____

____

____

"If you want us to call the police, what code words would you like to use? If we call the police, where would you like us to send them?"

____

____

"If at any point, you would like to contact us or have any questions or concerns, please call the confidential study hotline at: 555-555-5555" [give Women's Health Study card]

1. What is your phone number?

Home (__ __ __) __ __ __-__ __ __ __ Okay to leave a message? □ Yes □ No
Work (__ __ __) __ __ __-__ __ __ __ Okay to leave a message? □ Yes □ No
Cell (__ __ __) __ __ __-__ __ __ __ Okay to leave a message? □ Yes □ No Okay to text? □ Yes □ No

2. What is your current address? Okay to send newsletter? □ Yes □ No

____
____
____

"In the event that we are unable to get in touch with you we may, with your permission, contact people you trust who may be able to help us reach you. We will not share any information with anyone we contact about the nature of the study or information you share with use. Please provide contact information for anyone we could contact in an effort to reach you."

3. Contact Person 1

Name: ____ Relationship:____________________

Address:____ How should we identify ourselves when we call?

____ ______________________________
____ ______________________________

Phone # (__ __ __) __ __ __-__ __ __ __ Home ______________________________

(__ __ __) __ __ __-__ __ __ __ Work

(__ __ __) __ __ __-__ __ __ __ Cell

4. Contact Person 2

Name: ____ Relationship: ____________________

Address:____ How should we identify ourselves when we call?

____ ______________________________
____ ______________________________

Phone # (__ __ __) __ __ __-__ __ __ __ Home ______________________________

(__ __ __) __ __ __-__ __ __ __ Work

(__ __ __) __ __ __-__ __ __ __ Cell

5. Contact Person 3

Name: ____ Relationship: ____________________

Address:____ How should we identify ourselves when we call?

____ ______________________________
____ ______________________________

Phone # (__ __ __) __ __ __-__ __ __ __ Home ______________________________

(__ __ __) __ __ __-__ __ __ __ Work

(__ __ __) __ __ __-__ __ __ __ Cell

Note: it is helpful to let participants know about times when study personnel will be in the ED and how the participant can reach us. Tell the participant that she can call the ED and ask to speak with someone from the "SHIP Project." Encourage participants to call or drop in with new contact information or to check in at any time by calling the study hotline number 555-555-5555.
